# Genomic Variation and Phenotypic Diversity of Coffee-Derived *Lactiplantibacillus plantarum* Isolates

**DOI:** 10.3390/ijms27188307

**Published:** 2026-09-18

**Authors:** Gabriela N. Tenea, Victor Cifuentes, Jazmin Hidalgo

**Affiliations:** Biofood and Nutraceutics Research and Development Group, Faculty of Engineering in Agricultural and Environmental Sciences, Universidad Técnica del Norte, Av. 17 de Julio s-21 y José María Córdova, Ibarra 100150, Ecuador

**Keywords:** *Lactiplantibacillus plantarum*, coffee fermentation, whole-genome resequencing, genomic diversity, SNPs, strain-level variation, stress tolerance

## Abstract

*Lactiplantibacillus plantarum* exhibits substantial strain-level genomic diversity that may contribute to variation in stress tolerance and functional properties. Five isolates recovered from spontaneously fermented red coffee cherries were analyzed by whole-genome resequencing and phenotypic characterization. Single-nucleotide polymorphisms (SNPs) and short insertions and deletions (InDels) were identified and functionally annotated. SNPs predominated, ranging from 16,865 to 20,280 per isolate, with CATR21 showing the highest SNP and InDel counts. Synonymous variants were the most frequent predicted consequence, followed by upstream and missense variants, whereas high-impact variants were uncommon. Selected isolate-specific missense variants affected *atpF* and *atpB* in CATR7, *mobQ* in CATR14, *ptsP* in CATR21, and *glf* and *epsC* in TYPR1. Phenotypic responses were strain- and condition-dependent. CATR21 exhibited the highest viability at pH 2.5 and the highest 24-h autoaggregation among the coffee-derived isolates. CATR7 showed the highest viability under 1% NaCl and the highest numerical viability under 4% NaCl. Genome-wide variant counts did not show a consistent relationship with the evaluated phenotypic traits. Overall, the combined genomic and phenotypic analyses demonstrated substantial strain-level variability among the coffee-derived *L. plantarum* isolates across the evaluated conditions.

## 1. Introduction

Lactic acid bacteria (LAB) are important microorganisms in food fermentation and are increasingly investigated for their technological and functional properties. Their ability to modify fermentation substrates, produce organic acids and other metabolites, and interact with surrounding microbial communities contributes to food quality and microbial ecology [1,2].

Among LAB, *Lactiplantibacillus plantarum* exhibits substantial genomic plasticity and ecological versatility. Comparative genomic studies have revealed extensive strain-level variation among isolates from fermented foods, plant-associated environments, and other niches, including differences in genes involved in carbohydrate metabolism, stress responses, cell-surface properties, and other cellular functions [3,4,5,6]. However, genomic variation does not necessarily translate directly into phenotypic differences, as its functional effects depend on the genes affected, their regulatory context, and interactions among physiological pathways.

Whole-genome resequencing (WGR) provides a useful approach for characterizing strain-level genetic variation and identifying candidate variants that may contribute to bacterial adaptation. In a previous study, whole-genome resequencing of two native *L. plantarum* strains identified strain-specific genetic variation and candidate gene variants potentially associated with stress adaptation and antimicrobial properties [7]. These findings support genome-wide variant analysis as a complementary approach to phenotypic characterization, while emphasizing the need to distinguish sequence variation from experimentally demonstrated functional effects.

Coffee-cherry fermentation represents a particularly relevant ecological context for investigating strain-level diversity in *L. plantarum*. The mucilage surrounding coffee beans is rich in fermentable carbohydrates and undergoes rapid changes in sugar availability, organic-acid composition, pH, and microbial interactions during spontaneous fermentation [8,9]. Microorganisms persisting in this dynamic environment are therefore exposed to changing nutritional and physicochemical conditions that may favor substantial strain-level functional diversity. *L. plantarum* is particularly relevant because of its metabolic versatility and frequent occurrence in plant-associated and fermented-food environments. Characterizing genomic variation among coffee-derived isolates can therefore provide insight into the genetic diversity present within this fermentation-associated population and identify candidate functions for subsequent investigation of traits relevant to microbial persistence and fermentation performance. Nevertheless, whether individual genomic differences represent specific adaptations to the coffee environment cannot be established from comparative sequence analysis alone. LAB occurring in this environment may exhibit strain-dependent genomic and functional characteristics; however, whether these characteristics represent adaptation specifically to coffee fermentation remains unknown [10]. In previous work, LAB isolated from spontaneously fermented coffee cherries exhibited strain-dependent antimicrobial, redox, and immunomodulatory properties [11]. However, genomic variation among coffee-associated *L. plantarum* isolates and its relationship to selected functional phenotypes remains insufficiently characterized.

To address this gap, five *L. plantarum* isolates recovered from spontaneously fermented red coffee cherries were comparatively investigated using WGR and phenotypic characterization. Genomic analysis focused on SNPs and InDels, their predicted functional consequences, shared and variable genomic positions among isolates, and the identification of isolate-specific missense variants. Selected loci carrying isolate-specific missense variants were prioritized for qualitative interpretation based on variant occurrence and the annotated functions of their encoded proteins. Phenotypic characterization included viability under acidic, bile, and NaCl stress, together with cell-surface hydrophobicity (CSH), autoaggregation, and cholesterol reduction in the culture medium. Genomic and phenotypic profiles were evaluated as complementary measures of strain diversity, with an exploratory assessment of associations between genome-wide variant metrics and selected phenotypic traits.

## 2. Results

### 2.1. Sequencing Quality and Mapping Performance

After adapter and quality trimming, the sequence data comprised 3.27–3.48 Gb and 21.77–23.17 million reads per isolate. The trimmed reads had GC contents of 43.3–44.2%, Q20 values of 99.7%, and Q30 values of 97.1–97.3% (Table S1). After quality trimming with Trimmomatic, reads were mapped to the *L. plantarum* ST-III reference genome, with 78.8–90.09% of reads mapping to the reference (Table 1). Genome coverage at ≥1× ranged from 90.87% to 92.09%, and mean sequencing depth ranged from 730× to 890×. The number of detected PASS variants, including SNPs and InDels, ranged from 17,576 to 21,172 among the isolates (Table 1).

### 2.2. Genome-Wide Identification and Distribution of Sequence Variants

**Figure 1:**
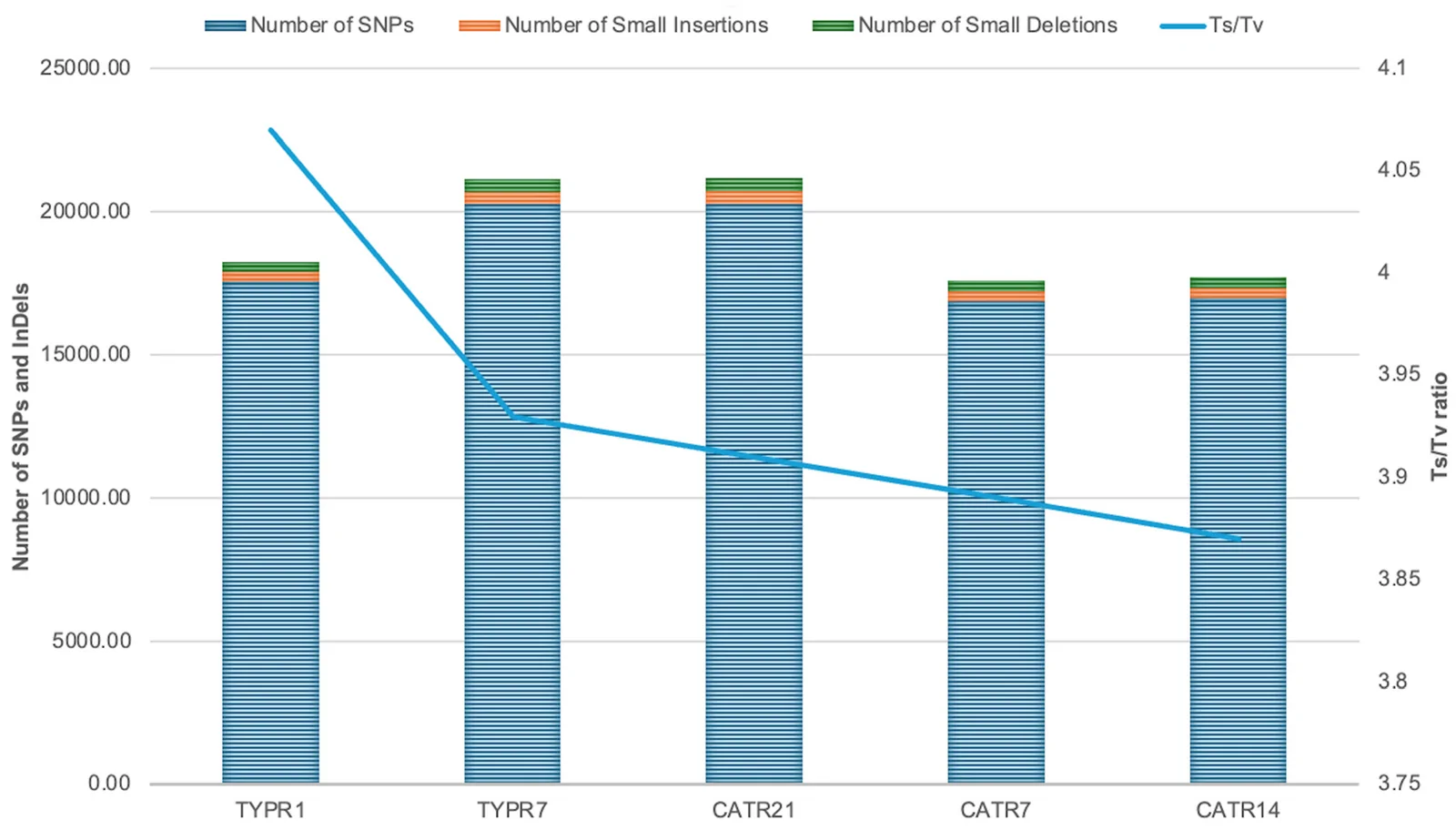
Genome-wide sequence variants identified in the five coffee-derived *L. plantarum* isolates relative to the ST-III reference genome. Bars show the numbers of SNPs and InDels; the line shows the transition/transversion ratio plotted on the secondary axis.

Genome-wide variant calling against the *L. plantarum* ST-III reference genome identified SNPs as the predominant variant class in all five coffee-derived isolates (Figure 1). CATR21 and TYPR7 had the largest SNP counts, whereas CATR7 and CATR14 had the lowest. InDels were detected at substantially lower frequencies than SNPs, with CATR21 showing the highest number of both variant types. The Ts/Tv ratios were similar among isolates (3.87–4.07), with transitions occurring more frequently than transversions. Genome-wide mapping of the variants showed that SNPs and InDels were distributed across the chromosome, with local differences in variant density among isolates (Figure S2). The comparable Ts/Tv ratios and chromosome-wide distribution of variants indicate a similar overall substitution pattern among the isolates, while differences in variant abundance reflect strain-level sequence variation relative to the ST-III reference genome.

### 2.3. Functional Annotation and Predicted Impact of Genomic Variants

Functional annotation of the retained PASS variant dataset showed a similar distribution of predicted consequences among the five coffee-derived *L. plantarum* isolates (Table 2). According to the SnpEff impact classification, most annotated variants belonged to categories not predicted to cause major disruption of protein function. Synonymous variants, classified as LOW impact, represented 54.4–54.9% of annotations, whereas upstream-gene variants, classified as MODIFIER, accounted for 22.0–22.8% (Table 2). Missense variants, classified as MODERATE impact, represented 20.7–21.5% and constituted the predominant category involving amino-acid substitutions. In contrast, HIGH-impact consequences were uncommon: frameshift variants represented only 0.78–1.02%, while stop-gained and start-lost variants each occurred at frequencies below 0.2%. Thus, although substantial sequence variation was detected relative to the ST-III reference genome, variants predicted to substantially disrupt coding sequences represented only a small fraction of the annotated variant set (Table S2). The relative distribution of these consequence categories was consistent across isolates, despite differences in the total number of variants. Missense variants accounted for approximately one-fifth of the annotations and therefore represented the principal category of predicted protein-altering substitutions considered in the subsequent strain-level analysis.

### 2.4. Comparative Analysis of Shared Genomic and Isolate-Specific Missense Variants

Comparison of the five coffee-derived *L. plantarum* isolates identified 5043 reference-genome coordinates containing a variant record in all five isolate VCF files. Of these, 4955 (98.3%) contained the same REF/ALT allele combination in all isolates, whereas 88 (1.7%) showed allelic differences among isolates. Isolate-specific missense variants were identified by comparing variant coordinates and alternate alleles across the five coffee-derived isolates. All missense variants selected in Table 3 were represented by single-allele genotype calls in the corresponding isolate datasets. In CATR14, *mobQ*, encoding a putative molybdate transport-associated protein, carried seven isolate-specific codon-level amino-acid changes: p.His39Arg, p.Ala60Glu, p.Asn64Asp, p.Gln67Lys, p.Thr147Ser, p.Lys155Glu, and p.Ile157Val. In CATR7, the selected loci included two genes encoding components of the F_0_ ATP synthase complex. The *atpF* gene, encoding subunit b, carried five codon-level amino-acid changes (p.Glu84Asp, p.Lys91Gln, p.Ser98Ala, p.Ser99Thr, and p.Ala102Ser), whereas *atpB*, encoding subunit a, carried p.Ile17Val and p.Ile21Phe. In CATR21, *ptsP*, encoding phosphoenolpyruvate-protein phosphotransferase PtsP, was selected based on the p.Pro311Leu substitution. In TYPR1, the selected loci were *glf*, encoding UDP-galactopyranose mutase and carrying seven missense substitutions (p.Ser128Ile, p.His221Asn, p.Asp223Asn, p.Tyr229Phe, p.Val233Met, p.Asp243Asn, and p.Leu249Phe), and *epsC*, encoding an exopolysaccharide biosynthesis-associated protein and carrying the p.Leu45Phe substitution. No locus from TYPR7 met the applied selection criteria.

### 2.5. Tolerance to Simulated Gastrointestinal and Osmotic Stress Conditions

Cell viability differed among strains under the simulated gastrointestinal and osmotic stress conditions evaluated (Table 4A,B). No significant differences were observed among strains in the controls after 4 h, whereas small strain-dependent differences were detected in the 24-h controls. Under simulated gastric conditions, CATR21 showed the highest viability at pH 2.5, whereas LP and CATR7 showed the lowest and second-lowest values, respectively. At pH 3.0, TYPR1 had the highest numerical viability, although it did not differ significantly from CATR21. The relative performance of the isolates therefore varied between the two acidic conditions. Bile exposure also resulted in significant strain-dependent differences at both concentrations (*p* < 0.05). At 0.3% bile, CATR7 showed the highest viability, whereas LP showed the lowest. At 1% bile, CATR21 exhibited significantly higher viability than the other strains, while LP again showed the lowest value. Significant strain-dependent differences were also observed at both NaCl concentrations (*p* < 0.05). At 1% NaCl, CATR7 showed the highest viability overall, whereas CATR14 showed the lowest. At 4% NaCl, LP showed the highest viability overall. Among the coffee-derived isolates, CATR7 had the highest numerical viability at 4% NaCl, although it did not differ significantly from TYPR1; CATR14 again showed the lowest viability. Overall, strain performance was condition-dependent: CATR21 performed best at pH 2.5 and 1% bile, CATR7 at 0.3% bile and 1% NaCl, LP at 4% NaCl, and TYPR1 showed the highest numerical viability at pH 3.0. These phenotypic differences were subsequently examined in relation to the genomic variation identified among the coffee-derived isolates.

### 2.6. Cell-Surface Properties

**Figure 2:**
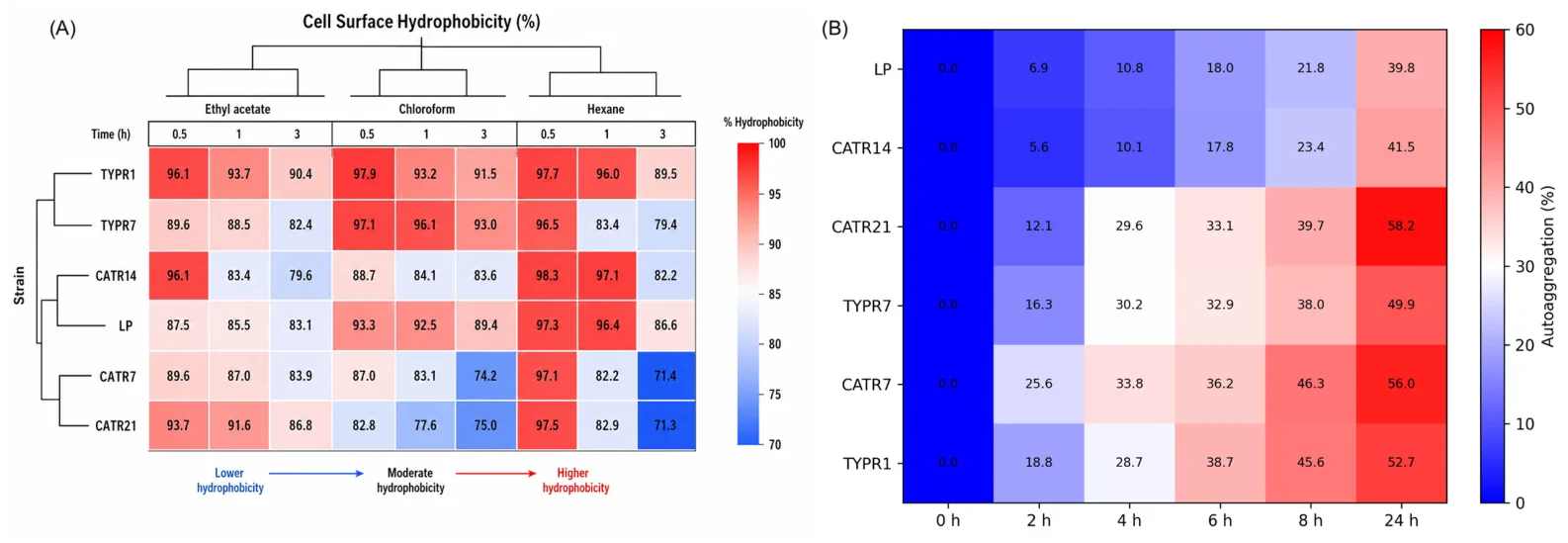
(**A**) Heatmap with hierarchical clustering showing the cell-surface hydrophobicity (%) of *L. plantarum* strains toward ethyl acetate, chloroform, and hexane after 0.5, 1, and 3 h of incubation. Cell values indicate hydrophobicity percentages; clustering was based on Euclidean distance using the complete-linkage method. (**B**) Autoaggregation profiles of the *L. plantarum* strains. Values represent the percentage of autoaggregation at each incubation time. LP, reference strain *L. plantarum* ATCC 8014.

Cell-surface hydrophobicity varied among the *L. plantarum* isolates according to solvent and incubation time (Figure 2A). Hydrophobicity remained above 70% across all evaluated conditions, although the magnitude and temporal pattern of variation differed among isolates. TYPR1 exhibited consistently high hydrophobicity across the three solvents, with final values of 90.4% for ethyl acetate, 91.5% for chloroform, and 89.5% for hexane. TYPR7 also maintained high hydrophobicity, particularly with chloroform, with values of 97.1%, 96.1%, and 93.0% at 0.5, 1, and 3 h, respectively. In contrast, CATR21 and CATR7 showed pronounced time-dependent decreases, particularly in chloroform and hexane. CATR21 decreased from 82.8% to 75.0% in chloroform and from 97.5% to 71.3% in hexane, whereas CATR7 decreased from 87.0% to 74.2% and from 97.1% to 71.4%, respectively. CATR14 exhibited high initial hydrophobicity toward ethyl acetate and hexane but showed marked decreases after 3 h. The reference strain (LP) displayed relatively modest changes with ethyl acetate and chloroform, while hydrophobicity toward hexane decreased from 97.3% at 0.5 h to 86.6% at 3 h. Among the solvents, hexane produced the highest initial hydrophobicity values across all strains, ranging from 96.5% to 98.3%. Overall, the temporal changes in hydrophobicity were strain- and solvent-dependent.

Autoaggregation generally increased over time, although the magnitude and kinetics differed among isolates (Figure 2B). CATR7 showed the most rapid increase during the early incubation period, reaching 25.6% at 2 h, 33.8% at 4 h, and 46.3% at 8 h. TYPR1 showed the highest autoaggregation among the strains at 6 h (38.7%) and reached a value like that of CATR7 at 8 h (45.6%). By 24 h, CATR21 exhibited the highest level among the coffee-derived isolates (58.2%), followed by CATR7 (56.0%), TYPR1 (52.7%), and TYPR7 (49.9%). CATR14 showed the slowest increase, reaching 41.5%, compared with 39.8% for the reference strain. Overall, the isolates displayed distinct aggregation profiles, with CATR7 characterized by rapid early development and CATR21 by the highest final level.

### 2.7. Cholesterol Reduction in the Culture Medium

Significant strain-dependent differences were observed in both cholesterol reduction and survival following incubation in cholesterol-supplemented medium (Table 5; *p* < 0.05). The reference strain LP exhibited the highest cholesterol reduction, followed by CATR7 and TYPR7, whereas CATR21 showed markedly lower removal than all other strains. Among the coffee-derived isolates, CATR7 displayed the highest cholesterol reduction. Survival showed a different strain distribution. CATR21 exhibited the highest survival, followed by TYPR1 and CATR7, whereas TYPR7 showed lower survival and LP the lowest value overall. The strain rankings for cholesterol reduction and survival were therefore discordant. LP combined the highest cholesterol reduction with the lowest survival, while CATR21 showed the opposite pattern, with the highest survival but the lowest cholesterol reduction. Thus, cholesterol reduction from the culture medium was not directly reflected by survival under the cholesterol-supplemented condition, indicating that these phenotypes varied among strains independently of their relative survival during incubation.

### 2.8. Exploratory Correlations Between Genomic Variation and Phenotypic Traits

**Figure 3:**
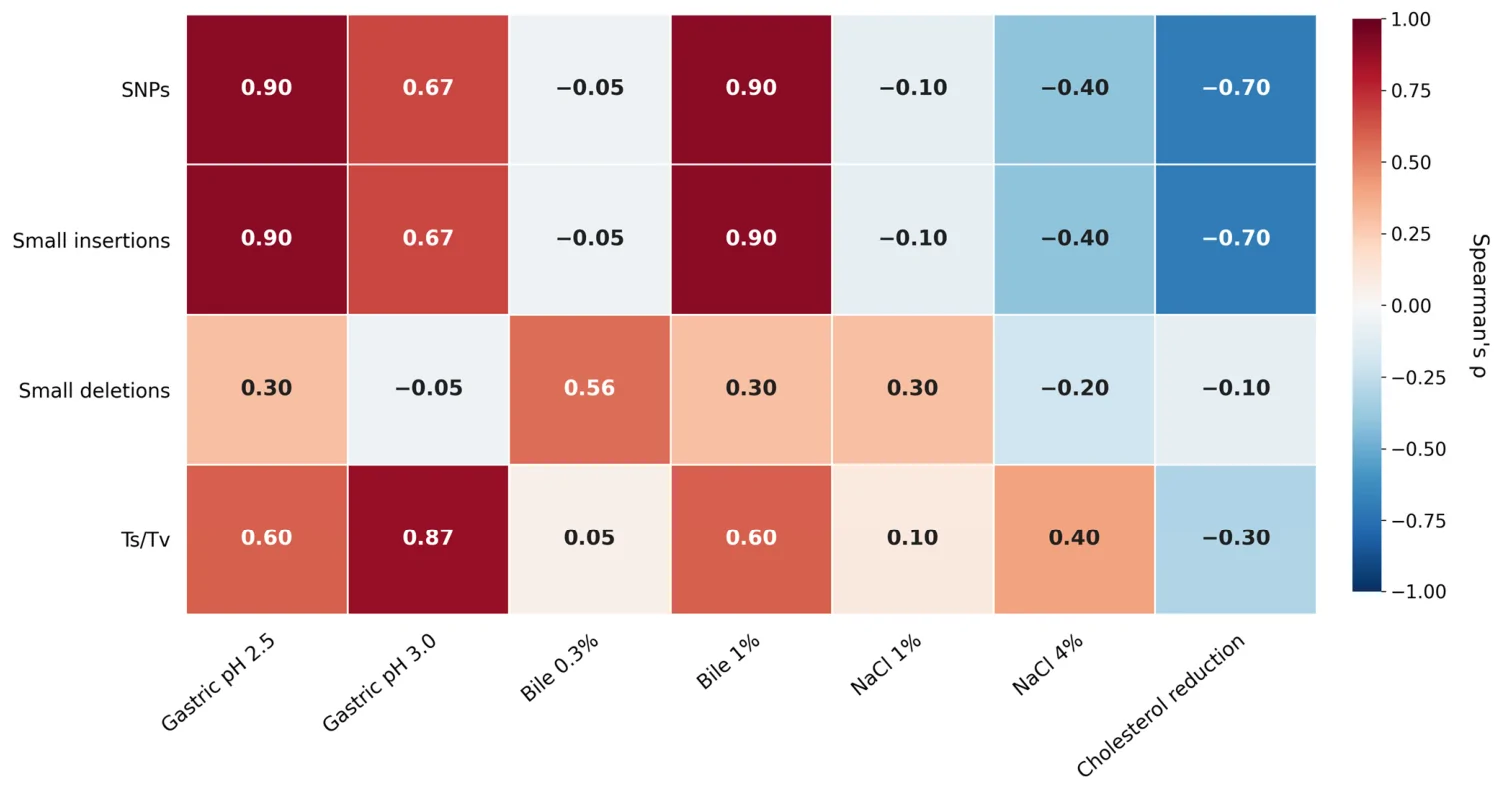
Exploratory associations between genome-wide variant counts and selected phenotypic traits among the five coffee-derived *L. plantarum* isolates. Coefficients were calculated from strain-level mean values. Positive and negative correlations are shown in red and blue, respectively. Because only five isolates were included, the analysis was exploratory and no inferential conclusions were drawn.

Spearman rank correlation analysis was used to explore relationships between genome-wide variant metrics and selected phenotypic measurements among the five coffee-derived isolates (Figure 3). SNP and small-insertion counts produced identical correlation patterns because their rankings across isolates were the same. Both metrics showed large positive correlations with viability at pH 2.5 and under 1% bile exposure (ρ = 0.90), as well as a moderate positive correlation with viability at pH 3.0 (ρ = 0.67). Conversely, SNP and small-insertion counts were negatively correlated with cholesterol reduction (ρ = −0.70) and viability under 4% NaCl (ρ = −0.40), whereas their correlations with viability under 0.3% bile and 1% NaCl were weak (ρ = −0.05 and −0.10, respectively). Small-deletion counts showed a moderate positive correlation with viability under 0.3% bile (ρ = 0.56), while their correlations with the remaining phenotypic measurements ranged from −0.20 to 0.30. The transition/transversion (Ts/Tv) ratio showed its largest positive correlation with viability at pH 3.0 (ρ = 0.87), followed by viability at pH 2.5 and under 1% bile exposure (ρ = 0.60 for both). Its correlations with the other phenotypic measurements ranged from −0.30 to 0.40. Because the analysis included only five isolates and was based on genome-wide summary metrics, these correlations were considered exploratory and descriptive. They were used to identify patterns for future investigation and do not establish statistically supported or causal genotype–phenotype relationships.

## 3. Discussion

The five coffee-derived *L. plantarum* isolates showed substantial nucleotide-level variation relative to the ST-III reference genome, with 16,865–20,280 SNPs detected per isolate (Table 1; Figure 1). This range was comparable to the 17,610 and 21,906 SNPs previously identified in *L. plantarum* UTNGt2 and UTNGt21A, respectively, using the reference genome applied in that study [7]. The Ts/Tv ratios obtained here (3.87–4.07) were also similar to those reported for these strains (3.82–3.96), suggesting broadly comparable substitution patterns between the datasets. Synonymous variants represented the predominant predicted consequence, whereas missense variants were less frequent and predicted high-impact effects were uncommon. This distribution is consistent with previous genomic analyses of *L. plantarum* [7] and with the extensive genomic plasticity reported among strains from diverse ecological niches [3,4,5]. Overall, the observed variation primarily reflected synonymous and other lower-impact changes rather than predicted disruption of protein-coding sequences. Comparison of the five isolate VCF files identified 5043 variant positions shared across all isolates. Of these, 4955 carried the same allelic state, whereas 88 showed allelic differences among isolates. The predominance of identical alleles at shared variant positions indicates that the isolates possess a substantial set of sequence differences relative to ST-III in common, together with a smaller subset of positions contributing to their differentiation. This comparison concerns positions containing variant records in all five VCF files and should not be interpreted as an analysis of the entire core genome.

The isolate-specific missense analysis provided a more focused view of the genomic differences among isolates (Table 3). In CATR7, isolate-specific missense variants were identified in *atpF* and *atpB*, which encode subunits of the F_0_ sector of the F_0_F_1_-ATP synthase complex. This system contributes to proton translocation, energy conservation, and intracellular pH homeostasis during acid stress [12]. Despite these variants, CATR7 showed relatively low viability at pH 2.5 (Table 4A), indicating that their presence was not associated with increased acid tolerance under the conditions tested. Missense substitutions in ATP synthase components cannot be assumed to enhance protein function, as their effects may be neutral, detrimental, or dependent on the genetic and physiological background [13]. Acid tolerance in *L. plantarum* involves multiple cellular processes, including ATPase activity, membrane composition, metabolic regulation, and other acid-stress response mechanisms. Therefore, the *atpF* and *atpB* variants identified in CATR7 should be regarded as candidate strain-specific genomic features rather than determinants of enhanced acid tolerance. Their functional effects could be further evaluated through gene-expression analysis, ATPase activity measurements, and targeted allelic replacement.

In CATR14, seven isolate-specific codon-level amino-acid changes were identified in *mobQ* (Table 3). This gene is annotated as encoding a putative molybdate transport-associated protein, although no evident relationship exists between this function and the phenotypes evaluated in the present study. Moreover, CATR14 did not show enhanced viability under acid, bile, or NaCl stress and had the lowest viability under both NaCl concentrations (Table 4A,B). The presence of several substitutions within one gene therefore does not, by itself, provide evidence of functional adaptation.

The p.Pro311Leu substitution in *ptsP* identified in CATR21 represents another potentially relevant isolate-specific difference. PtsP participates in phosphotransferase-system-associated cellular regulation, while carbohydrate utilization and its regulatory networks are known to vary considerably among *L. plantarum* strains [14]. However, the phenotype of CATR21 was condition-dependent: it showed the highest viability at pH 2.5 and 1% bile but did not exhibit the highest viability under NaCl stress or the greatest cholesterol reduction (Table 4A,B and Table 5). Consequently, the *ptsP* substitution provides a candidate for further investigation but cannot be assigned a broad functional effect from the present results.

In TYPR1, *glf* encodes UDP-galactopyranose mutase and is associated with carbohydrate metabolism, which is highly variable among *L. plantarum* strains [14]. The substitution in *epsC* is also of interest because exopolysaccharide-associated functions may influence cell-envelope characteristics, including hydrophobicity and aggregation. These surface properties vary among *L. plantarum* strains and may be affected by extracellular polysaccharides and other cell-envelope components [15]. Nevertheless, TYPR1 carried other isolate-specific variants, and the present analysis does not permit its cell-surface phenotype to be attributed specifically to the p.Leu45Phe substitution in *epsC*.

The phenotypic responses were strongly dependent on the condition tested (Table 4A,B). No coffee-derived isolate consistently performed best under acid, bile, and NaCl exposure. CATR21 performed best at pH 2.5 and 1% bile, whereas CATR7 showed the highest viability at 0.3% bile and 1% NaCl. At 4% NaCl, LP had the highest viability overall, while CATR7 had the highest numerical value among the coffee-derived isolates but did not differ significantly from TYPR1. Such variability is expected because tolerance to environmental stress in *L. plantarum* involves interacting mechanisms related to proton homeostasis, ATP metabolism, membrane properties, transport systems, and broader stress-response pathways [13,16]. The contrasting responses observed among the isolates therefore probably reflect the combined activity of multiple physiological systems rather than the effect of a single sequence variant.

Differences were also evident in the cell-surface assays (Figure 2A). Hydrophobicity varied according to isolate, solvent, and incubation time, whereas autoaggregation followed distinct temporal patterns (Figure 2B). The survival observed for several coffee-derived isolates under pH 2.5–3.0 and 0.3% bile is consistent with the broad range of acid- and bile-tolerance phenotypes previously reported among candidate probiotic *L. plantarum* strains isolated from fermented foods and other sources [17]. However, direct numerical comparison across studies is limited by differences in exposure time, medium composition, inoculum density, and viability endpoints. The present results therefore support strain-specific robustness under the conditions tested rather than equivalence to previously characterized probiotic strains [18]. Although these characteristics may influence bacterial interactions with their environment, the present data do not support linking any specific variant to the observed hydrophobicity or autoaggregation profiles.

Cholesterol reduction was strain-dependent and was not proportional to survival in cholesterol-supplemented medium. The reference strain showed the greatest cholesterol reduction despite the lowest survival, whereas CATR21 showed the opposite pattern, and CATR7 combined relatively high cholesterol reduction with high survival. This discordance suggests that cholesterol removal may involve mechanisms beyond active metabolism by viable cells, including cell-surface adsorption, membrane incorporation, and bile-associated processes [19]. Although hydrophobicity provides information on cell-surface properties, its strain-dependent pattern did not parallel cholesterol reduction and therefore does not provide direct evidence of cholesterol binding. Together, these findings indicate that cholesterol removal is likely influenced by multiple strain-specific cellular and metabolic properties. Studies in animal systems further indicate that the persistence and biological effects of beneficial microorganisms are strongly dependent on the microbial strain and host context [20,21].

The exploratory correlations between genome-wide variant metrics and phenotypic measurements should be interpreted cautiously because only five coffee-derived isolates were included (Figure 3), limiting statistical power and making Spearman coefficients sensitive to individual strain rankings. The observed associations are therefore considered descriptive and hypothesis-generating. Moreover, genome-wide variant counts do not account for variant type, predicted functional impact, or the biological roles of affected loci. Thus, these analyses do not establish statistically supported or causal genotype–phenotype relationships and require validation in a larger strain collection.

Because *L. plantarum* is haploid, the approximately 4–5% of mixed-allele calls observed under the diploid computational model are unlikely to represent conventional biological heterozygosity. These signals may instead reflect low-frequency variation within the cultured population, ambiguous or reference-dependent read mapping, or sequencing and variant-calling noise. Their origin cannot be resolved from the present data, and they were therefore not considered evidence of fixed strain-level variation. The selected changes in *atpF*, *atpB*, *mobQ*, *ptsP*, *glf*, and *epsC* provide candidates for subsequent functional investigation (Table 3, Table 4 and Table 5; Figure 2 and Figure 3). Targeted mutagenesis, gene-expression analysis, or comparative functional studies will be required to determine whether these substitutions contribute to the observed phenotypes.

Taken together, the present study focused on genomic variation and selected *in vitro* phenotypes and did not characterize metabolite production during coffee fermentation. Because genomic variation alone cannot establish how strain-level differences influence fermentation-associated metabolism, integration of genomics with metabolomic profiling represents an important next step. Comparative analysis of intracellular and extracellular metabolites, particularly those associated with carbohydrate utilization, organic-acid metabolism, amino-acid metabolism, and other fermentation-related pathways, could help determine whether the genomic differences identified here correspond to distinct metabolic outputs. Such multi-omics integration, ideally under coffee-derived fermentation conditions, would provide a stronger basis for linking strain-level genomic diversity with functional roles during coffee fermentation.

## 4. Materials and Methods

### 4.1. Bacterial Strains

Five *L. plantarum* isolates were recovered from spontaneously fermented red coffee cherries (*Coffea arabica* L.) representing two cultivars: Typica Red (TYPR1 and TYPR7) and Red Caturra (CATR7, CATR14, and CATR21). The isolates were selected from a larger collection based on preliminary functional screening, including antimicrobial activity against foodborne pathogens and tolerance to acidic conditions. Taxonomic identification was performed by 16S rRNA gene sequencing, and the resulting sequences were compared against sequences available in the NCBI GenBank nucleotide database using the NCBI BLASTn web service (National Center for Biotechnology Information, Bethesda, MD, USA; accessed on 15 January 2026).

The corresponding GenBank accession numbers were PZ868889 for TYPR1, PZ868888 for TYPR7, PZ868890 for CATR7, PZ868891 for CATR14, and PZ868892 for CATR21. Isolates were maintained at −80 °C in de Man, Rogosa and Sharpe (MRS) broth (Difco, Detroit, MI, USA) supplemented with 20% (*v*/*v*) glycerol. *L. plantarum* ATCC 8014 (LP) was included as the control strain in the phenotypic assays.

### 4.2. WGR, Quality Assessment, and Read Mapping

Genomic DNA from the five isolates was analyzed by whole-genome resequencing using an Illumina platform with paired-end 151-bp reads. Sequence quality was assessed using FastQC v0.11.7 [22], including per-base sequence quality, GC content, and the presence of adapter sequences. Adapter sequences and low-quality bases were removed using Trimmomatic v0.38 [23] with the following parameters: ILLUMINACLIP:Adapter.fa:2:30:10:8:true, LEADING:15, TRAILING:15, SLIDINGWINDOW:4:15, and MINLEN:36. The complete *L. plantarum* ST-III reference assembly (GenBank assembly accession GCF_000148815.2) [24] was used for read mapping and variant identification. Filtered paired-end reads were aligned to the reference genome using BWA-MEM v0.7.17-r1188 [25]. Alignment files were processed in BAM format, and duplicate reads were identified using Picard MarkDuplicates and excluded from downstream variant analysis. Mapping performance was evaluated based on the proportion of mapped reads, the number and percentage of reference positions covered at ≥1×, and mean sequencing depth.

### 4.3. Variant Calling and Filtering

Variant calling was performed using the Genome Analysis Toolkit (GATK) v4.2.0.0 [26]. Following generation of individual genomic VCF (GVCF) files, variants were genotyped against the *L. plantarum* ST-III reference assembly using GenotypeGVCFs. Genotyping was performed with a sample-ploidy setting of 2 (--sample-ploidy 2) and a minimum phred-scaled calling-confidence threshold of 30 (--standard-min-confidence-threshold-for-calling 30.0). SNPs were hard-filtered using the following criteria: quality by depth (QD) < 2.0, Fisher strand bias (FS) > 60.0, strand odds ratio (SOR) > 3.0, root-mean-square mapping quality (MQ) < 40.0, mapping-quality rank-sum test (MQRankSum) < −12.5 and read-position rank-sum test (ReadPosRankSum) < −8.0. Short InDels were filtered using QD < 2.0, FS > 200.0, and ReadPosRankSum < −20.0. Only variants marked PASS in the resulting VCF files were retained for downstream annotation and comparative analyses. Because *L. plantarum* has a haploid genome, genotype classifications generated under the diploid computational setting were not interpreted as biological ploidy states. Mixed-allele calls classified as heterozygous by the original workflow were retained in the PASS dataset but were not considered individually as evidence of fixed strain-level variation. Accordingly, homozygous and heterozygous designations were treated solely as computational genotype classifications generated by the original GATK workflow.

### 4.4. Variant Annotation and Genomic Characterization

Variants marked PASS were characterized according to their reference-genome coordinates and nucleotide substitutions. SNPs were classified as transitions (A↔G and C↔T) or transversions, and the transition/transversion (Ts/Tv) ratio was calculated for each isolate. Functional annotation was performed using SnpEff v5.0e [27] based on the annotation of the *L. plantarum* ST-III reference assembly. Variants were assigned Sequence Ontology terms and classified according to their predicted impact as HIGH, MODERATE, LOW, or MODIFIER. Predicted consequences, including synonymous, missense, frameshift, stop-gained, start-lost, upstream-gene, downstream-gene, and intergenic variants, were recorded. When a variant had multiple predicted consequences, the first-ranked consequence reported by SnpEff, representing the predicted most deleterious effect, was used for classification. Variant coordinates were visualized along the ST-III chromosome to examine their genomic distribution. All annotated effects were considered *in silico* predictions and were not interpreted as experimental evidence of altered gene function or phenotype.

#### 4.4.1. Comparative Analysis of Shared Genomic Variants

Variant records were compared among the five isolates according to chromosome and position in the reference genome. A position was defined as a shared variant coordinate when a variant was detected at that position in the variant call format (VCF) file of every isolate. At each shared coordinate, the reference allele (REF) and alternate allele or alleles (ALT) were compared among the five isolates. A shared coordinate was classified as allelically identical only when all five VCF files contained the same REF and ALT allele combination. Coordinates containing different ALT alleles among isolates were classified as shared coordinates with allelic variation. Candidate loci were selected based on the occurrence of multiple isolate-specific missense variants within the same coding sequence and/or an annotated function potentially relevant to the phenotypes evaluated. These comparisons were used only to identify candidates for subsequent investigation; no causal genotype–phenotype association was inferred because the functional effects of the individual amino acid substitutions were not experimentally validated.

#### 4.4.2. Identification of Isolate-Specific Missense Variants

Annotated variants were compared across the five isolates to identify isolate-specific coding changes. An isolate-specific allele was defined as an alternate allele present at a reference-genome position in only one isolate. When different alternate alleles occurred at the same position, each allele was evaluated independently. SNPs annotated as missense variants were retained, whereas synonymous variants were excluded. For each isolate-specific missense variant, the genomic position, nucleotide substitution, predicted amino acid change, affected gene, and annotated protein function were recorded. Candidate loci were selected when multiple isolate-specific missense variants occurred within the same coding sequence and/or when the annotated gene function was relevant to the phenotypes examined. All missense variants selected for further interpretation were represented by single allele calls in the corresponding isolate datasets. These variants were considered candidate genomic differences, as their functional effects were not experimentally validated and no causal genotype–phenotype relationships were inferred.

### 4.5. In Vitro Characterization of Probiotic-Associated Phenotypes of the Candidate Isolates

#### 4.5.1. Simulated Gastrointestinal Stress Tolerance

Tolerance to simulated gastric conditions and bile salts was evaluated based on the method described by Alizadeh Behbahani et al. [28], with modifications to include two acidic conditions. Briefly, overnight cultures of each isolate were harvested by centrifugation at 5000× *g* for 5 min at 4 °C, washed twice with sterile Ringer’s solution (pH 7.2), and resuspended in simulated gastric juice. The basal gastric solution contained glucose (3.5 g/L), NaCl (2.05 g/L), KH_2_PO_4_ (0.60 g/L), CaCl_2_ (0.11 g/L), and KCl (0.37 g/L). Immediately before use, lysozyme (0.1 g/L), porcine bile (0.05 g/L), and pepsin (13.3 mg/L) were added according to Corcoran et al. [29]. The gastric pH values were modified to 2.5 and 3.0 for the present study. Cell suspensions were incubated at 37 °C for 4 h. Viable cell counts were determined at 0 and 4 h by serial dilution and plating on MRS agar, followed by incubation at 37 °C for 48 h. Results were expressed as log CFU/mL. Bile salt tolerance was evaluated independently at 0.3% and 1.0% (*w*/*v*) oxgall. Overnight cultures were inoculated into MRS broth supplemented with the corresponding oxgall concentration at an initial concentration of approximately 8 log CFU/mL and incubated at 37 °C for 4 h. Viable cell counts were determined at 0 and 4 h by serial dilution and plating on MRS agar, followed by incubation at 37 °C for 48 h, and expressed as log CFU/mL. Parallel controls were incubated for 4 h under the same conditions in MRS broth without added oxgall or simulated gastric components. LP was included as the reference strain. All assays were performed using three independent biological cultures.

#### 4.5.2. Sodium Chloride Tolerance

Sodium chloride tolerance was evaluated by inoculating overnight cultures of each isolate into MRS broth supplemented with 1% or 4% (*w*/*v*) NaCl. Cultures were incubated at 37 °C for 24 h. Viable cell concentrations were determined by serial dilution and plating on MRS agar, followed by incubation at 37 °C for 48 h, and expressed as log CFU/mL. The two NaCl concentrations were selected to represent separated levels of osmotic challenge rather than to establish a continuous NaCl dose–response curve. Accordingly, the assay was intended to compare strain viability under moderate and stronger NaCl exposure. Parallel controls were incubated for 24 h in MRS broth without additional NaCl. LP was included as a reference strain. All assays were performed using three independent biological cultures.

#### 4.5.3. Cell Surface Hydrophobicity (CSH) and Autoaggregation

CSH and autoaggregation were determined according to Liang et al. [30] with minor modifications. For the hydrophobicity assay, bacterial cells were harvested by centrifugation (5000× *g*, 5 min, 4 °C), washed twice with phosphate-buffered saline (PBS, pH 7.2), and resuspended to approximately 1 × 10^8^ CFU/mL. The initial absorbance (A_0_) was measured at 600 nm. Equal volumes of the bacterial suspension and either hexane, chloroform, or ethyl acetate were mixed by vortexing for 1 min and allowed to separate at room temperature. After mixing and phase separation, hydrophobicity was determined at 0.5, 1, and 3 h of incubation at room temperature by measuring the absorbance of the aqueous phase (A_1_) at 600 nm. Hydrophobicity was calculated as: Hydrophobicity (%) = [1 − (A_1_/A_0_)] × 100. For the autoaggregation assay, bacterial cultures (approximately 8 log CFU/mL) were centrifuged (5000× *g*, 15 min, 4 °C), washed twice with PBS, and resuspended in the same buffer. After vortexing for 10 s, the initial absorbance (A_0_) was recorded at 600 nm. Suspensions were then incubated statically at 37 °C, and absorbance was measured at 2, 4, 6, 8, and 24 h. Autoaggregation was calculated as: Autoaggregation (%) = [1 − (A_t_/A_0_)] × 100. All assays were performed using three independent biological cultures. LP was used as reference strain. Mean hydrophobicity values were analyzed by hierarchical clustering based on Euclidean distance and the complete-linkage method [31].

#### 4.5.4. Cholesterol Removal in the Culture Medium

The ability of the isolates to reduce cholesterol concentration in the culture medium was evaluated using a modified colorimetric method based on Rudel and Morris [32], as adapted by Tomaro-Duchesneau et al. [33]. Overnight bacterial cultures were adjusted to an initial OD_600_ of 1.0 and inoculated into MRS broth supplemented with cholesterol at 100 μg/mL. Parallel uninoculated MRS broth supplemented with cholesterol served as the control for calculating cholesterol reduction. Cultures were incubated at 37 °C for 24 h, after which viable cell concentrations were determined by serial dilution and plating on MRS agar. Survival under the cholesterol-supplemented condition was calculated relative to the initial viable cell concentration as: Survival (%) = (final viable cell concentration/initial viable cell concentration) × 100. Following incubation, cultures were centrifuged at 4000× *g* for 10 min at 4 °C, and 500 μL of cell-free supernatant was transferred to a clean tube. An equal volume of 33% (*w*/*v*) KOH was added, followed by 1 mL of cold absolute ethanol. Samples were incubated at 37 °C for 15 min, cooled to room temperature, and mixed with 1 mL of sterile distilled water and 1.5 mL of hexane. After vortexing and phase separation, 500 μL of the upper hexane phase was transferred to a clean tube and evaporated to dryness. The residue was dissolved in 3 mL of o-phthalaldehyde solution (50 mg/dL), followed by addition of 750 μL of concentrated sulfuric acid. After incubation at room temperature for 20 min, absorbance was measured at 570 nm. Cholesterol concentrations were determined from a calibration curve generated using known cholesterol standards, and sample concentrations were calculated using the corresponding regression equation. Cholesterol reduction was calculated as the difference between the cholesterol concentration measured in the uninoculated control and that remaining in the corresponding bacterial culture after 24 h.

### 4.6. Statistical Analysis

Data are presented as mean ± standard deviation (SD) from three independent biological replicates. For each experimental condition, differences among strains were evaluated using the Kruskal–Wallis test followed by Dunn’s multiple-comparison test. Statistical significance was set at *p* < 0.05. To explore relationships between genomic variation and phenotypic characteristics, Spearman’s rank correlation coefficients (ρ) were calculated between genome-wide variant metrics (SNP, insertion, deletion counts, Ts/Tv) and selected phenotypic measurements, including viability under simulated gastric, bile, NaCl stress conditions and cholesterol reduction in the culture medium. Spearman correlations between genomic variant metrics and phenotypic traits were calculated using the five coffee-derived isolates only. The observed rank correlations should therefore be interpreted as descriptive patterns within this small strain set rather than robust estimates of population-level associations.

## 5. Conclusions

The five coffee-derived *Lactiplantibacillus plantarum* isolates exhibited substantial strain-level genomic and phenotypic diversity. Although CATR21 had the highest overall variant burden, genome-wide variant abundance did not consistently correspond to performance across the phenotypic conditions evaluated. Isolate-specific missense variants were identified in candidate loci including *atpF*, *atpB*, *mobQ*, *ptsP*, *glf*, and *epsC*. These variants provide targets for future functional investigation, but the present data do not establish causal genotype–phenotype relationships. Overall, the findings demonstrate distinct combinations of genomic variation and functional traits among coffee-derived *L. plantarum* isolates and provide a strain-resolved basis for subsequent experimental validation.

## Figures and Tables

**Table 1:** Read-mapping performance and genome-wide variant counts for the five coffee-derived isolates.

Sample ID	Reference Positions Covered (≥1×)	Reference Genome Coverage (%)	Total Reads	Mapped Reads (%)	Mean Depth	PASS Annotated Variants
TYPR1	3,046,348	92.09	22,828,924	90.09	890	18,240
TYPR7	3,039,751	91.89	23,172,834	79.50	787	21,144
CATR21	3,039,979	91.89	21,765,388	78.80	730	21,172
CATR7	3,005,945	90.87	21,891,294	89.95	844	17,576
CATR14	3,009,500	90.97	22,081,376	85.76	809	17,697

Sample ID, sample name; reference positions covered (≥1×), number of mapped reference-genome positions; reference genome coverage (%), percentage of the reference genome covered by mapped reads; total reads, combined number of Read 1 and Read 2 sequences; mapped reads (%), percentage of reads mapped to the reference genome; mean depth, average alignment depth; PASS-annotated variants, SNP and short-InDel calls marked PASS and retained in the original variant dataset for functional annotation.

**Table 2:** Functional annotation and predicted impact of genomic variants identified in five *L. plantarum* isolates from coffee-cherry fermentations relative to the reference strain ST-III.

Variant Annotation	Description	Predicted Impact on Gene Function *	TYPR1 (%)	TYPR7 (%)	CATR21 (%)	CATR7 (%)	CATR14 (%)
synonymous_variant	Codon change that does not alter the encoded amino acid	LOW	54.84	54.90	54.66	54.50	54.41
upstream_gene_variant	Variant located upstream of a gene (default distance: 5 kb)	MODIFIER	22.85	22.49	22.64	21.97	22.22
missense_variant	Codon change that results in a different amino acid	MODERATE	20.67	20.74	20.82	21.47	21.38
frameshift_variant	Insertion or deletion that alters the translational reading frame	HIGH	0.78	1.01	1.01	1.02	0.95
downstream_gene_variant	Variant located downstream of a gene (default distance: 5 kb)	MODIFIER	0.19	0.17	0.17	0.23	0.23
disruptive_inframe_deletion	In-frame deletion that changes one codon and removes one or more codons	MODERATE	0.15	0.14	0.08	0.13	0.11
disruptive_inframe_insertion	In-frame insertion that changes one codon and adds one or more codons	MODERATE	0.12	0.00	0.08	0.07	0.07
stop_gained	Variant that introduces a premature stop codon	HIGH	0.11	0.10	0.10	0.15	0.16
conservative_inframe_insertion	In-frame insertion of one or more codons	MODERATE	0.08	0.08	0.08	0.09	0.09
conservative_inframe_deletion	In-frame deletion of one or more codons	MODERATE	0.09	0.00	0.09	0.08	0.07
splice_region_variant&stop_retained_variant	Variant annotated as affecting a splice region while retaining the stop codon	LOW	0.06	0.07	0.07	0.13	0.12
stop_lost&splice_region_variant	Variant that removes a stop codon and is annotated within a splice region	HIGH	0.04	0.03	0.03	0.05	0.05
frameshift_variant&stop_gained	Frameshift variant associated with the introduction of a premature stop codon	HIGH	0.03	0.04	0.04	0.03	0.03
start_lost	Variant that changes the start codon into a non-start codon	HIGH	0.02	0.01	0.01	0.03	0.03
frameshift_variant&stop_lost&splice_region_variant	Frameshift variant associated with stop-codon loss and a splice-region annotation	HIGH	0.01	0.00	0.00	0.02	0.00
stop_gained&conservative_inframe_insertion	In-frame insertion associated with the introduction of a premature stop codon	HIGH	0.01	0.00	0.00	0.00	0.00
gene_fusion	Variant annotated as producing the fusion of two genes	HIGH	0.00	0.00	0.01	0.01	0.01
frameshift_variant&splice_region_variant	Frameshift variant associated with a splice-region annotation	LOW	0.00	0.00	0.01	0.01	0.02
stop_lost	Variant that changes a stop codon into a non-stop codon	HIGH	0.00	0.00	0.00	0.00	0.01
initiator_codon_variant	Variant that changes the start codon into another start codon encoding a different amino acid	LOW	0.00	0.00	0.00	0.00	0.01

* HIGH, variants predicted by SnpEff to have a potentially severe effect on gene function; MODERATE, variants predicted to alter the encoded protein sequence without necessarily abolishing function; LOW, variants predicted to have limited effects on protein sequence; MODIFIER, variants located outside coding sequences or in regions for which the functional effect is difficult to predict. SnpEff impact categories are computational predictions and do not constitute experimental evidence of altered gene function.

**Table 3:** Selected isolate-specific missense variants in candidate genes among coffee-derived *L. plantarum* isolates.

Isolate	Candidate Gene	Encoded Protein/Function	Predicted Amino-Acid Changes
CATR14	*mobQ*	Putative molybdate transport-associated protein	p.His39Arg, p.Ala60Glu, p.Asn64Asp, p.Gln67Lys, p.Thr147Ser, p.Lys155Glu, p.Ile157Val
CATR7	*atpF*	F_0_ ATP synthase subunit b	p.Glu84Asp, p.Lys91Gln, p.Ser98Ala, p.Ser99Thr, p.Ala102Ser
CATR7	*atpB*	F_0_ ATP synthase subunit a	p.Ile17Val, p.Ile21Phe
CATR21	*ptsP*	Phosphoenolpyruvate-protein phosphotransferase PtsP	p.Pro311Leu
TYPR1	*Glf*	UDP-galactopyranose mutase	p.Ser128Ile, p.His221Asn, p.Asp223Asn, p.Tyr229Phe, p.Val233Met, p.Asp243Asn, p.Leu249Phe
TYPR1	*epsC*	Exopolysaccharide biosynthesis-associated protein	p.Leu45Phe

Only selected isolate-specific missense variants are presented; selection does not imply a demonstrated association with the measured phenotypes.

**Table 4:** Viability of *Lactiplantibacillus plantarum* strains under simulated gastrointestinal and osmotic stress conditions.

**(A). Cell Viability (log_10_ CFU/mL)**					
Strain	Control (4 h)	0.3% Bile (4 h)	1% Bile (4 h)	Gastric Juice, pH 2.5 (4 h)	Gastric Juice, pH 3.0 (4 h)
CATR14	10.53 ± 0.01 ^a^	7.70 ± 0.03 ^d^	7.55 ± 0.07 ^c^	6.60 ± 0.05 ^c^	7.65 ± 0.03 ^c^
CATR21	10.54 ± 0.01 ^a^	8.48 ± 0.01 ^b^	8.25 ± 0.04 ^a^	7.61 ± 0.01 ^a^	8.45 ± 0.02 ^ab^
CATR7	10.54 ± 0.01 ^a^	8.95 ± 0.01 ^a^	7.54 ± 0.02 ^c^	3.88 ± 0.15 ^d^	7.65 ± 0.06 ^c^
LP	10.54 ± 0.01 ^a^	6.71 ± 0.04 ^e^	6.58 ± 0.02 ^d^	3.28 ± 0.02 ^e^	4.17 ± 0.04 ^d^
TYPR1	10.57 ± 0.02 ^a^	8.31 ± 0.07 ^c^	8.02 ± 0.04 ^b^	7.01 ± 0.03 ^b^	8.51 ± 0.03 ^a^
TYPR7	10.55 ± 0.01 ^a^	8.48 ± 0.03 ^b^	7.56 ± 0.02 ^c^	6.61 ± 0.03 ^c^	8.33 ± 0.03 ^b^
**(B). Cell Viability (log_10_ CFU/mL)**					
Strain	Control (24 h)	1% NaCl (24 h)	4% NaCl (24 h)		
CATR14	10.54 ± 0.03 ^ab^	5.75 ± 0.03 ^d^	5.52 ± 0.09 ^d^		
CATR21	10.54 ± 0.02 ^ab^	8.45 ± 0.02 ^b^	6.53 ± 0.02 ^c^		
CATR7	10.56 ± 0.07 ^a^	9.45 ± 0.01 ^a^	7.45 ± 0.01 ^b^		
LP	10.41 ± 0.01 ^c^	8.44 ± 0.06 ^b^	8.68 ± 0.04 ^a^		
TYPR1	10.50 ± 0.02 ^abc^	7.80 ± 0.03 ^c^	7.33 ± 0.02 ^b^		
TYPR7	10.52 ± 0.07 ^abc^	7.53 ± 0.04 ^c^	6.94 ± 0.02 ^c^		

Cell viability is expressed as log CFU/mL (Data are presented as mean ± SD from three biological replicates). Panel A presents viability after 4 h of incubation exposure to bile salts or simulated gastric juice and control. Panel B presents viability after 24 h of incubation in MRS broth without additional NaCl (control) or in MRS broth supplemented with 1% or 4% (*w*/*v*) NaCl. Different lowercase superscript letters within each column indicate significant differences among strains according to the Kruskal–Wallis test followed by Dunn’s multiple-comparison test (*p* < 0.05). LP, reference strain *L. plantarum* ATCC 8014.

**Table 5:** Cholesterol reduction in the culture medium (µg/mL) and survival (%) of *L. plantarum* strains after incubation in cholesterol-supplemented medium.

Strain	Cholesterol Reduction in the Culture Medium (µg/mL)	Survival (%)
TYPR1	3.10 ± 0.01 ^d^	86.04 ± 0.05 ^b^
TYPR7	5.36 ± 0.25 ^c^	74.55 ± 0.09 ^e^
CATR7	6.49 ± 0.02 ^b^	85.57 ± 0.01 ^c^
CATR14	3.36 ± 0.02 ^d^	81.03 ± 0.01 ^d^
CATR21	0.53 ± 0.02 ^e^	89.59 ± 0.01 ^a^
*L. plantarum* ATCC 8014 (LP)	7.53 ± 0.01 ^a^	54.81 ± 0.01 ^f^

Survival was calculated from viable cell counts before and after incubation. Values are presented as mean ± standard deviation (n = 3). Different lowercase letters within each column indicate significant differences among strains according to Dunn’s multiple-comparison test following the Kruskal–Wallis test (*p* < 0.05).

## Data Availability

The datasets generated and analyzed during the current study are included in this published article and its Supplementary Materials. The VCF files have been deposited in the Zenodo repository (10.5281/zenodo.22021297).

## Supplementary Materials

The following supporting information can be downloaded at https://www.mdpi.com/article/10.3390/ijms27188307/s1.
