# Supplementary material for: Genomic Variation and Phenotypic Diversity of Coffee-Derived Lactiplantibacillus plantarum Isolates
Source: Int J Mol Sci. 2026 Sep 18;27(18):8307. doi: 10.3390/ijms27188307 (PMC13607025; doi:10.3390/ijms27188307)

Supplementary materials

Table S1. Sequencing quality and mapping statistics

Sample ID	Total Bases (bp)	Total Reads	GC (%)	AT (%)	Q20 (%)	Q30 (%)
TYPR1	3,426,879,476	22,828,924	44.2	55.8	99.7	97.2
TYPR7	3,478,830,225	23,172,834	43.4	56.6	99.7	97.2
CATR21	3,267,657,875	21,765,388	43.3	56.7	99.7	97.1
CATR7	3,285,968,905	21,891,294	44.1	55.9	99.7	97.3
CATR14	3,314,624,946	22,081,376	44.1	55.9	99.7	97.1

•Sample ID : Sample name.
• Total Bases (bp) : Total number of bases sequenced.
• Total Reads : Total number of reads. For Illumina paired-end sequencing, this value refers to the sum of read1 and read2.
• GC (%) : Ratio of GC content.
• AT (%) : Ratio of AT content.
• Q20 (%) : Ratio of bases that have phred quality score of over 20.
• Q30 (%) : Ratio of bases that have phred quality score of over 30.

Table S2. Condensed distribution of predicted variant consequences identified by SnpEff in the five coffee-derived *L. plantarum* isolates.

Sample ID	CATR14	CATR21	CATR7	TYPR1	TYPR7
upstream_gene_variant	3,933	4,793	3,862	4,168	4,756
downstream_gene_variant	41	36	41	34	37
frameshift_variant	169	213	179	142	213
missense_variant	3,784	4,409	3,774	3,771	4,386
synonymous_variant	9,629	11,573	9,579	10,003	11,697
intergenic_region	0	0	0	0	0
stop_lost	1	0	0	0	0
start_lost	6	3	6	3	3
stop_gained	29	22	27	20	21
5′ UTR_variant	0	0	0	0	0
3′ UTR_variant	0	0	0	0	0
splice_acceptor_variant	0	0	0	0	0
splice_donor_variant	0	0	0	0	0
Others	105	123	107	99	121

Sample ID : Sample name
• upstream_gene_variant : Upstream of a gene (default length: 5K bases)
• downstream_gene_variant : Downstream of a gene (default length: 5K bases)
• frameshift_variant : Insertion or deletion causes a frame shift
• missense_variant : Variant causes a codon that produces a different amino acid
• synonymous_variant : Variant causes a codon that produces the same amino acid
• intergenic_region : The variant is in an intergenic region
• stop_lost : Variant causes stop codon to be mutated into a non-stop codon
• start_lost : Variant causes start codon to be mutated into a non-start codon.
• stop_gained : Variant causes a stop codon
• 5_prime_UTR_variant : Variant hits 5'UTR region
• 3_prime_UTR_variant : Variant hits 3'UTR region
• splice_acceptor_variant : The variant hits a splice acceptor site (defined as two bases before exon start, except for the first exon)
• splice_donor_variant : The variant hits a splice donor site (defined as two bases after coding exon end, except for the last exon)

"Others" comprises low-frequency consequence categories not presented separately in this condensed summary. Counts represent predicted SnpEff annotations and may not correspond directly to the numbers of unique variants reported in Table 1.

Figure S1. Resequencing workflow

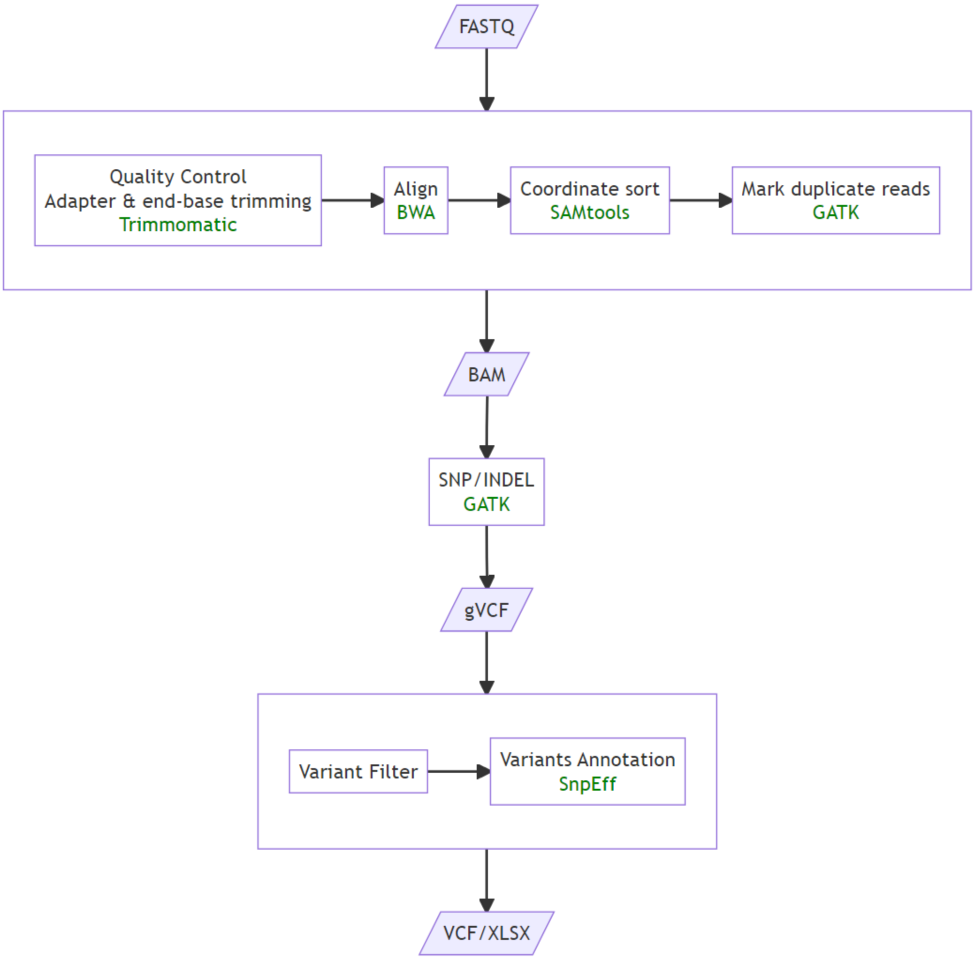

Figure S2. Circular genome maps showing the chromosomal distribution of genomic variants identified in the five *L. plantarum* strains relative to the reference genome ST-III. From the outer to the inner rings, tracks represent chromosome coordinates, single nucleotide polymorphisms (SNPs), small insertions, small deletions, GC content, and GC skew. Positive GC skew values are shown in green and negative values in purple.

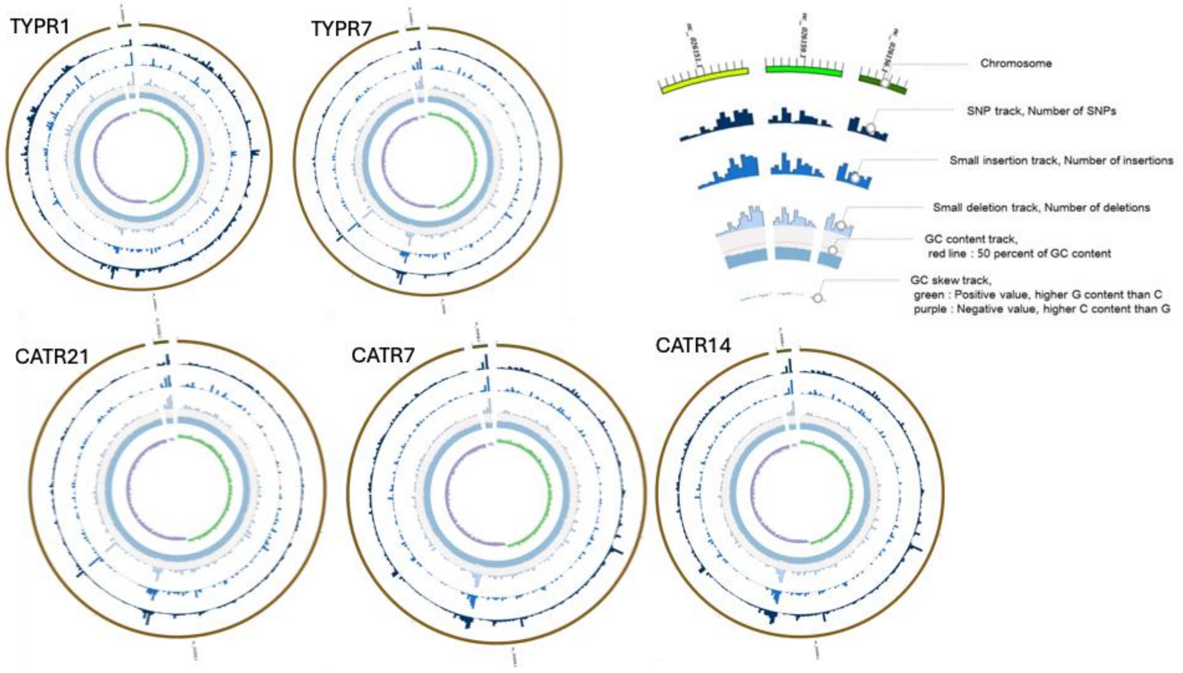

Supplement: Supplementary file 1 [file ijms-27-08307-s001.zip › ijms-4564980-supplementary.pdf]
